# Implementing nursing best practice guidelines: Impact on patient referrals

**DOI:** 10.1186/1472-6955-6-4

**Published:** 2007-06-28

**Authors:** Nancy Edwards, Barbara Davies, Jenny Ploeg, Tazim Virani, Jennifer Skelly

**Affiliations:** 1 School of Nursing and Department of Epidemiology and Community Medicine, University of Ottawa, Ottawa, Canada; 2 School of Nursing, University of Ottawa, Ottawa, Canada; 3 School of Nursing, McMaster University, Hamilton, Canada; 4 Registered Nurses' Association of Ontario, Toronto, Ontario, Canada; 5 School of Nursing, McMaster University, Hamilton, Canada

## Abstract

**Background:**

Although referring patients to community services is important for optimum continuity of care, referrals between hospital and community sectors are often problematic. Nurses are well positioned to inform patients about referral resources. The objective of this study is to describe the impact of implementing six nursing best practice guidelines (BPGs) on nurses' familiarity with patient referral resources and referral practices.

**Methods:**

A prospective before and after design was used. For each BPG topic, referral resources were identified. Information about these resources was presented at education sessions for nurses. Pre- and post-questionnaires were completed by a random sample of 257 nurses at 7 hospitals, 2 home visiting nursing services and 1 public health unit. Average response rates for pre- and post-implementation questionnaires were 71% and 54.2%, respectively. Chart audits were completed for three BPGs (n = 421 pre- and 332 post-implementation). Post-hospital discharge patient interviews were conducted for four BPGs (n = 152 pre- and 124 post-implementation).

**Results:**

There were statistically significant increases in nurses' familiarity with resources for all BPGs, and self-reported referrals to specific services for three guidelines. Higher rates of referrals were observed for services that were part of the organization where the nurses worked. There was almost a complete lack of referrals to Internet sources. No significant differences between pre- and post-implementation referrals rates were observed in the chart documentation or in patients' reports of referrals.

**Conclusion:**

Implementing nursing BPGs, which included recommendations on patient referrals produced mixed results. Nurses' familiarity with referral resources does not necessarily change their referral practices. Nurses can play a vital role in initiating and supporting appropriate patient referrals. BPGs should include specific recommendations on effective referral processes and this information should be tailored to the community setting where implementation is taking place.

## Background

The provision of continuous, comprehensive client care is critical in today's complex health care system where clients experience shorter hospital stays, and move quickly from one care setting to another. Continuity of care is an essential feature of a well-functioning health care system. It is demarcated by coherent and linked services and results from "good information flow, good interpersonal skills, and good coordination of care" [1]. Achieving continuity of care requires a seamless referral system [2]. Nurses can play a pivotal role to help ensure that referral processes function well. Best practice guidelines (BPGs) that include evidence-based recommendations for appropriate referrals may improve continuity of care.

The referral process is a systematic approach to help clients use services or resources, with the aims of promoting wellness, and enhancing self-care and quality of care [2]. There is considerable evidence, primarily in the medical literature, that referral processes in health care can be improved [3]. For example, comprehensive cardiac rehabilitation reduces mortality and morbidity, but these programs are used by only a fraction of eligible patients and by far fewer women than men [4,5]. A review of 32 studies of cardiac rehabilitation found that the main predictor of referrals was the physician's endorsement of the effectiveness of such a program [6]. Study findings indicate that there is significant, unexplained variation in referral rates of general practitioners and that factors related to patient, practice and general practitioner characteristics explain no more than half of this variation.

A limited number of studies [7-10] have explored nursing referral processes and identified challenges in their implementation. Townsend et al. [8] examined referrals made by liaison public health nurses of 570 high-risk multiparous women discharged from postpartum units. Although nurses used a standardized form identifying high-risk criteria, there was poor agreement between risk factors identified by liaison and district public health nurses. Risk factors most frequently missed at the time of hospital discharge included single or handicapped parent, family history of neglect or abuse, smoking parent and financial problems. A survey of 75 nurses in a home care agency [7] found that they resisted referring clients to hospice care because they felt they could adequately provide services and they desired to maintain patient continuity within their agency. However, late referrals made it difficult for the hospice to provide the full range of services possible and reduced potential cost savings to families and the health care system. Further, many nurses had limited knowledge about the hospice program. In a qualitative study of referral practices by primary care nurse practitioners to secondary care [10], study participants discussed the difficulties in making nursing referrals due to issues associated with professional boundary changes such as teamwork, communication, and professional relationships. Nurses talked about the value of guidelines for making appropriate referrals and the importance of taking on responsibility as gatekeepers to health resources.

Recommendations for appropriate and timely referrals are included in many clinical practice guidelines [11-13]. While studies examining the implementation of clinical practice guidelines have frequently assessed clinical outcomes [14,15], they have less often examined patterns of client referrals. We found one systematic review of interventions to improve outpatient referrals from primary care physicians to secondary or specialist care [3]. This review of 17 studies concluded that passive dissemination of local referral guidelines, feedback of referral rates, and discussion with an independent medical advisor were ineffective strategies. However, disseminating guidelines with structured referral sheets and involving consultants in educational activities were generally effective strategies to improve referral processes.

## Methods

### Research objective

The Registered Nurses' Association of Ontario has been developing and implementing nursing best practice guidelines in the province of Ontario, Canada since 2001 [16]. Our team led the provincial evaluation of this initiative, using a prospective before and after study to examine the impact of implementing nursing clinical best practice guidelines on nursing care practices, and patient outcomes. This article describes findings from cycle 3, implemented during the period 2003–4, and addresses one of our research questions: Were there changes in referral outcomes following implementation of six BPGs (asthma, breastfeeding, delirium-dementia-depression, smoking cessation, venous leg ulcers and diabetes foot care)?

### Intervention

The BPG implementation process is described in detail elsewhere [17]. The guidelines include a number of recommendations regarding client referrals. Referral sources identified were national associations, Internet websites and types of service providers or regional programs. Given the lack of research on the effectiveness of referral processes, most of the recommendations reflected levels three or four evidence. Level three evidence is that obtained from well-designed non-experimental descriptive studies, such as correlational studies. Level four evidence includes expert committee reports or opinion, and clinical experience of respected authorities. Levels three and four evidence was used only when directly applicable studies of good quality were not found during an extensive search of the literature. Illustrative examples of referral recommendations included in the BPGs are shown in Table 1.

**Table 1:** Examples of recommendations pertaining to referrals from RNAO BPGs

**BPG and Reference**	**Selected examples of recommendations pertaining to referrals**
Adult Asthma Care** RNAO, 2004	Assessment of Asthma Control: 1.3 For individuals identified as potentially having uncontrolled asthma, the level of acuity needs to be assessed by the nurse and an appropriate medical referral provided, i.e. urgent care or follow-up appointment.
	Referrals: 5.0 The nurse will facilitate referrals as appropriate. 5.1 Clients with poorly controlled asthma should be referred to their physician. 5.2 All clients should be offered links to community resources. 5.3 Clients should be referred to an asthma educator in their community, if appropriate and available.
Screening for Delirium, Dementia and Depression in Older Adults** RNAO, 2003a	Practice Recommendations: 7. When the nurse determines the client is exhibiting features of delirium, dementia and/or depression, a referral for a medical diagnosis should be made to specialized geriatric services, specialized geriatric psychiatry services, neurologists, and/or members of the multidisciplinary team, as indicated by screening findings. 8. Nurses should screen for suicide ideation and intent when a high index of suspicion for depression is present, and seek an urgent medical referral. Further, should the nurse have a high index of suspicion for delirium, an urgent medical referral is recommended.
Integrating Smoking Cessation into Daily Nursing Practice** RNAO, 2003b	Practice Recommendations: 4. Nurses should be knowledgeable about community smoking cessation resources, for referral and follow-up.

** Reprinted with permission of RNAO. The full set of BPGs and guideline recommendations is available on the RNAO website [13].

Health care organizations in Ontario were invited to participate in the implementation of a BPG through a request for proposals process. The implementing organizations were selected by peer review. At each site, a clinical resource nurse (CRN) led implementation of the BPG recommendations [16,18]. CRNs used a multi-strategy approach including educational sessions with staff nurses, reviews of policies and procedures with administrators and staff nurses, and modeling new clinical skills such as implementing and demonstrating the utility of new assessment tools or providing smoking cessation counseling. A standard toolkit [18] was used by nurses to guide their BPG implementation activities. Regular teleconference calls with the RNAO program director provided an opportunity for CRNs to address problem issues and to share their implementation strategies. CRNs tailored their implementation strategies to the organizational context, the patient population needs and the clinical gaps as assessed by their organization through patient satisfaction surveys and quality assurance programs.

### Participants

Eligibility criteria for participants were registered nurse, licensed practical nurse or health care aide; assigned to work on one of the units implementing the BPGs; and, not expected to go on maternity leave or to take an extended leave of absence from the clinical unit or from the agency during the six month period of guideline implementation. Patient eligibility criteria were developed for each BPG based on knowledge of the patient population characteristics at each site (see Table 2).

**Table 2:** Patient eligibility criteria for BPGs and participating program units

**BPG**	**Patient Eligibility Criteria **	**Participating Program Units**
Asthma	Patients who present to the participating units with a primary or secondary diagnosis of asthma	Emergency, in-patient and medical units
Breastfeeding	Postpartum women and their infants with a singleton birth, gestational age of 37 or more weeks, hospital length of stay for mother and infant 24 hours or more after birth, infant not delivered by a midwife. Additional infant criteria included: no major congenital anomalies, no galactasemia. Additional postpartum women criteria included: no documented use of street drugs, no major psychiatric illness, baby not up for adoption.	Labour and delivery, postpartum, neonatal intensive care, and public health unit
Delirium-Dementia-Depression	Clients admitted to participating units, age 65 years and older.	Surgical, medical and rehabilitation units
Smoking Cessation	Clients age 18 years and older admitted to participating unit. For outpatients, admission to out-patient services within the past month and currently attending the clinic.	In-patient and out-patient long-term mental health, in-patient unit for drug and alcohol addition, in-patient unit for acute psychiatric disorders, resource unit for nicotine dependency
Venous Leg Ulcers	All clients with new and recurrent venous leg ulcers. Patients with primary lymphedema, vasculitis, and under diabetic management excluded.	Chronic care unit, wound care clinic, and community care agency
Diabetes	Adults age 18 and over with a diagnosis of diabetes admitted to one of the participating units. Women with gestational diabetes excluded.	Oncology, medical and diabetes care and education centre and community care agency

### Data collection

Pre- and post-implementation data were obtained at each participating site with post-data collected 6 to 9 months after BPG implementation was initiated. Data were obtained from three sources: for all BPG implementation sites, a random sample of nurses on the participating units completed a self-administered questionnaire; for three BPGs (delirium-depression-dementia, smoking cessation and venous leg ulcer), a sequential sample of patient charts was retrieved from medical records for a period of six to eight weeks pre- and post-implementation; and, for four BPGs (asthma, breastfeeding, smoking cessation and diabetes foot care), patient interviews were completed by phone post-hospital discharge.

### Outcomes

A list of potential referral resources was developed for each BPG in consultation with the CRN and following a review of BPG recommendations. Specific referral resources were identified for the following: other members of the multi-disciplinary team, service provider organizations and information sources on self-management of the illness. In the pre- and post-implementation questionnaires, nurses were asked to rate their familiarity with relevant community resources on a scale of 1 to 5 (5 = extremely familiar, 1 = not familiar), and to indicate whether or not they had referred a patient to each of these resources in the past month (1 = yes, 0 = no or unsure). Since the number of referral resources listed varied by BPG, standardized mean scores and standard deviations were calculated. For the self-reported familiarity scale, standardized scores ranged from 1 to 5. Scores for self-reported referrals ranged from 0 to 1.

To assess whether patients had been given information about referral resources and whether or not they had telephoned, visited or received assistance from referral resources, patient interviews were conducted post-hospital discharge. For three of the BPGs (asthma, smoking cessation and diabetes foot care), patients were contacted at four weeks follow hospital discharge. For breastfeeding patients, follow-up data were collected at eight weeks postpartum.

For two topics, obtaining direct patient interview data post-discharge was not feasible due to either the nature of the patient population or service delivery issues. Specifically, with respect to the BPG of delirium-dementia-depression, concern was expressed about this vulnerable population as well as the complexity involved in obtaining consent from family members of participants with delirium or dementia. For the topic of venous leg ulcers, patient interviews were not feasible with the participating home visiting nursing service organization.

Chart abstraction forms were developed for each of the BPGs. However, we included extraction prompts for data on referrals for only three of the BPGs due to the absence of information about referrals to community resources in the hospitals charts for the other three topics (asthma, breast-feeding and diabetes foot care). The CRN conducted the first five to 10 pilot charts to make sure that the systems for obtaining the charts and the coding categories were feasible. Then she either trained another staff member to conduct the chart audit (delirium-dementia-depression and smoking cessation BPGs) or conducted the chart audits herself (venous leg ulcers BPG). Pilot data were included in the main findings.

For chart abstraction and patient interviews, documentation of a referral being made (from patient charts), or of a patient having been given information about a referral resource or having accessed a referral resource (from patient interviews) were coded dichotomously as follows: (1 = yes, 0 = no or don't know). An average score was computed across all referral resources listed for the specific BPG.

### Validity and reliability

Factor analysis, using varimax rotation was undertaken for the two referral measures (referral familiarity and referral practice) assessed by the nursing questionnaires for each BPG [19]. The Kaiser-Meyer-Olkin statistic was used to confirm sampling adequacy. Most factor analyses yielded single factor solutions for scale items. For referral familiarity items, eigen values ranged from 2.10 to 4.66; and for referral practice items, eigen values ranged from 1.47 to 3.04. Two scales yielded two factor solutions: resource familiarity for the venous leg ulcer and referral practices for the diabetes BPG. Each of these two-factor solutions differentiated between referral options within the organization (e.g. specialized clinics) and referral options external to the organization (e.g. community agencies). Cronbach's alpha for referral familiarity items ranged from .67 to .87, and for referral practices items ranged from .40 to .78 [19].

### Ethical considerations

The University of Ottawa health and social sciences ethics board provided ethics approval. Ethics reciprocity was requested and received from participating agencies. For the sites implementing the smoking cessation BPGs, the clinical data forms (chart audit, patient interviews) and the data collection protocol were also reviewed by their organizational ethics review board.

### Data analysis

Response rates for nurses were calculated based on the number of nurses randomly selected to participate in the study. In the analysis, we only included the responses of nurses who completed both pre- and post-implementation questionnaires. Patient follow-up interview response rates were estimated using the number of eligible patient charts as the denominator for the initial interviews conducted with patients while they were in hospital. The purpose of these initial interviews was to determine, from the client's perspective, whether nursing care received was consistent with the BPG recommendations selected by the organization for implementation. For the second set of follow-up interviews conducted by phone post-hospital discharge, response rates were calculated using the number of in-hospital interviews as the denominator. Patients were only asked about referrals in the second set of follow-up interviews.

Using paired t-tests, differences in mean scores between the pre- and post-implementation periods were compared for nurses' self-reported knowledge and referral patterns for each BPG. For the two scales with two-factor solutions, mean scores and pre- and post-implementation comparisons were computed for each factor. Pre- and post-implementation mean scores for documented patient referrals and patient's self-reports of referrals were compared using the independent student's t-test.

## Results

The BPGs were implemented in seven hospitals, two home visiting nursing service organizations and one public health unit. Seventeen program units participated in the pilot (see Table 2).

Response rates to the nurses' pre-implementation questionnaires ranged from 65.5% to 94.7% (average response rate 71%, see Table 3). Post-implementation, response rates ranged from 38.5% to 78.7% of the original sample (average response rate 54.2%). Some respondents answered only the pre-implementation questionnaires, while others answered only the post-implementation questionnaires. The 257 nurses answering both pre- and post-implementation questionnaires are included in this analysis. The majority of these nurses were female (93.7%), had been employed in nursing for more than six years (72.5%), and worked full-time (77.7%) as staff nurses (80.2%). About one third (35.3%) were baccalaureate or masters prepared. Just over half worked a combination of all three shifts (51.3%) with a small proportion working only nights and/or evenings (14.2%). There were no significant differences on these sociodemographic characteristics between nurses who responded to both questionnaires versus those who responded to only the pre- or post-implementation questionnaires. Response rates for chart audits were greater than 95% for all eligible cases. Response rates for follow-up interviews with patients ranged from 32.1% to 85.2% with an average response rate of 52.5%. Reasons for low response rates for patient interviews varied across BPGs and included language barriers, refusals, deaths, discharge to another facility rather than home, and failed efforts to contact patients. Administrative issues also affected patient recruitment and data collection for some BPGs, notably for asthma because we only had a part-time data collector during the day shift (and not on weekends); and for diabetes where there was a high turnover of CRN's and consequently, the follow-up of patients was inconsistent.

**Table 3:** Response rates for completion of nursing questionnaires, chart audits and patient interviews

	Asthma	Breastfeeding	DDD	Smoking Cessation	Venous Leg Ulcer	Diabetes Foot Care
**Pre-Implementation**						
# of nurse questionnaires	58	71	110	62	61	64
Questionnaire response rates	85.3%	94.7%	65.5%	70.5%	78.2%	68.1%
# of chart audits	80 ^1^	103 ^1^	196	116	109	98 ^1^
1^st ^patient interview (% of chart audits)	31 (38.8%)	75 (72.8%)	-	89 (76.7%)	-	66 (67.3%)
2^nd ^patient interview (% of 1^st ^interview)	21 (67.7%)	51 (49.5%)	-	42 (47.1%)	-	38 (57.6%)
**Post-Implementation**						
# of nurse questionnaires	38	59	97	46	30	55
Questionnaire response rates	55.9%	78.7%	57.7%	52.3%	38.5%	58.5%
# of nurses included in final analysis ^3^	34	53	67	41	25	37
Nurses with paired data (as % of random sample) ^3^	50.0%	70.7%	39.9%	46.6%	32.1%	39.4%
# of chart audits	48 ^1^	89 ^1^	187	93	52	123 ^1^
1^st ^patient interview (% of chart audits) ^2^	14 (29.2%)	80 (89.9%)	-	84 (90.3%)	-	58 (47.2%)
2^nd ^patient interview (% of 1^st ^interview)	10 (71.4%)	61 (85.2%)	-	27 (32.1%)	-	26 (44.8%)

Notes: ^1 ^No referral items included in chart audit

^2 ^No questions were included about referrals in the 1st in-hospital interview

^3 ^Both pre- and post-implementation questionnaires completed by nurses.

A comparison of pre- and post-BPG implementation responses to questionnaires indicates a statistically significant increase in nurses' familiarity with referral resources for all BPGs (see Table 4). For three of the six BPGs (asthma, delirium-depression-dementia and diabetes), an increase in the frequency of referrals was also reported by the nurses. However, pre- and post-measures of referral documentation in charts, and patient self-reports of referrals revealed no significant increases in overall referral rates.

**Table 4:** Summary of pre and post-implementation differences in referral outcomes by BPG

**BPG**		**Asthma **x¯ MathType@MTEF@5@5@+=feaafiart1ev1aaatCvAUfKttLearuWrP9MDH5MBPbIqV92AaeXatLxBI9gBaebbnrfifHhDYfgasaacH8akY=wiFfYdH8Gipec8Eeeu0xXdbba9frFj0=OqFfea0dXdd9vqai=hGuQ8kuc9pgc9s8qqaq=dirpe0xb9q8qiLsFr0=vr0=vr0dc8meaabaqaciaacaGaaeqabaqabeGadaaakeaacuWG4baEgaqeaaaa@2E3D@ (SD)	**Breast-feeding **x¯ MathType@MTEF@5@5@+=feaafiart1ev1aaatCvAUfKttLearuWrP9MDH5MBPbIqV92AaeXatLxBI9gBaebbnrfifHhDYfgasaacH8akY=wiFfYdH8Gipec8Eeeu0xXdbba9frFj0=OqFfea0dXdd9vqai=hGuQ8kuc9pgc9s8qqaq=dirpe0xb9q8qiLsFr0=vr0=vr0dc8meaabaqaciaacaGaaeqabaqabeGadaaakeaacuWG4baEgaqeaaaa@2E3D@ (SD)	**DDD **x¯ MathType@MTEF@5@5@+=feaafiart1ev1aaatCvAUfKttLearuWrP9MDH5MBPbIqV92AaeXatLxBI9gBaebbnrfifHhDYfgasaacH8akY=wiFfYdH8Gipec8Eeeu0xXdbba9frFj0=OqFfea0dXdd9vqai=hGuQ8kuc9pgc9s8qqaq=dirpe0xb9q8qiLsFr0=vr0=vr0dc8meaabaqaciaacaGaaeqabaqabeGadaaakeaacuWG4baEgaqeaaaa@2E3D@ (SD)	**Smoking Cessation **x¯ MathType@MTEF@5@5@+=feaafiart1ev1aaatCvAUfKttLearuWrP9MDH5MBPbIqV92AaeXatLxBI9gBaebbnrfifHhDYfgasaacH8akY=wiFfYdH8Gipec8Eeeu0xXdbba9frFj0=OqFfea0dXdd9vqai=hGuQ8kuc9pgc9s8qqaq=dirpe0xb9q8qiLsFr0=vr0=vr0dc8meaabaqaciaacaGaaeqabaqabeGadaaakeaacuWG4baEgaqeaaaa@2E3D@ (SD)	**Venous Leg Ulcer **x¯ MathType@MTEF@5@5@+=feaafiart1ev1aaatCvAUfKttLearuWrP9MDH5MBPbIqV92AaeXatLxBI9gBaebbnrfifHhDYfgasaacH8akY=wiFfYdH8Gipec8Eeeu0xXdbba9frFj0=OqFfea0dXdd9vqai=hGuQ8kuc9pgc9s8qqaq=dirpe0xb9q8qiLsFr0=vr0=vr0dc8meaabaqaciaacaGaaeqabaqabeGadaaakeaacuWG4baEgaqeaaaa@2E3D@ (SD)	**Diabetes Foot Care **x¯ MathType@MTEF@5@5@+=feaafiart1ev1aaatCvAUfKttLearuWrP9MDH5MBPbIqV92AaeXatLxBI9gBaebbnrfifHhDYfgasaacH8akY=wiFfYdH8Gipec8Eeeu0xXdbba9frFj0=OqFfea0dXdd9vqai=hGuQ8kuc9pgc9s8qqaq=dirpe0xb9q8qiLsFr0=vr0=vr0dc8meaabaqaciaacaGaaeqabaqabeGadaaakeaacuWG4baEgaqeaaaa@2E3D@ (SD)
**Nurses' Questionnaire**							
# of items – familiarity with referral source		5	5	3	5	2 + 7 ^1^	7
Nurses' familiarity with referral source (range 0–5)	Pre	2.03 (0.7)	2.51 (1.0)	3.33 (0.9)	2.13 (0.9)	4.14 (.81) 2.98 (.89)	2.59 (.78)
	Post	2.54 (0.9)**	2.74 (1.04)*	3.61 (1.1)*	2.9 (0.9)**	4.08 (.89) 3.78 (.85)**	3.13 (1.01)**
# of items – patient referrals made		4	3	3	3	9	3+4 ^1^
Patient referrals in past month by nurses (range 0–1)	Pre	0.23 (0.3)	0.20 (0.23)	0.29 (0.3)	0.22 (0.2)	0.28 (.26)	0.25 (.27) 0.17 (.20)
	Post	0.85 (.03)**	0.23 (0.24)	0.41 (0.4)*	0.25 (0.29)	0.36 (.26)	0.36 (.34) 0.32 (.30)**
**Chart Audit**							
# of referral sources		-	-	3	7	17	-
Referrals documented (range 0–1)	Pre	-	-	0.11 (.18)	0 (0)	0.15 (.08)	
	Post			0.13 (.22)	0.04 (.13)	0.17 (.07)	
**Patient Interviews**							
# of items – information about referral sources		6	11	-	7	-	8
Patient reports of having been given information in hospital about referral sources (range 0–1)	Pre	0.20 (.33)	0.44 (.18)	-	0.31 (.27)	-	0.12 (.13)
	Post	0.38 (.35)	0.47 (.18)		0.18 (.18)		0.13 (.18)
# of items – contact with referral sources		7	12				8
Patient reports of telephoning, visiting or receiving assistance from referral sources (range 0–1)	Pre	.23 (.13)	.12 (.07)	-	-	-	0.08 (.11)
	Post	.25 (.15)	.13 (.08)				0.09 (.10)

Notes: * p < .05

** p <.001

^1 ^Two-factor solutions were found. Pre- and post-implementation scores are provided for each of the factors.

There were some distinctive referral patterns reported by nurses, documented in the charts and ascertained through patient interviews. These included higher rates of referrals for services that were part of the organization where nurses were employed and almost a complete lack of referrals to Internet sources. In addition, for one of the BPGs (venous leg ulcers), the only statistically significant increase in referrals to individual resources observed on the chart audit was to other nurse specialists (e.g. enterostomal nurse or wound care specialist; 28.2% versus 50.6%, p < .0001).

## Discussion

Few previous studies have examined changes in referral patterns following implementation of nursing BPGs. We assessed changes in referrals from the perspective of both nurses and patients, as well as using chart audits. The patterns of reported referrals from these information sources offer important insights regarding the BPG implementation process and continuity of care.

Various factors may have influenced the uptake of referral recommendations. First, BPG recommendations for referrals are stated in rather general terms, written so as to be applicable to nurses working in various sectors of the health system. This lack of specificity may have limited their uptake. Second, content knowledge about the health issue is an important aspect of incorporating guideline recommendations into practice. For example the practice recommendations for delirium-depression-dementia in older adults require nurses to identify symptoms of delirium, dementia and depression in order to make a referral for medical diagnosis. The larger pre- versus post- intervention differences in nurses' familiarity scores relative to nurses' self-reported referrals was expected, as awareness about a referral source must precede decisions to make referrals to that source. Third, health care providers need more than knowledge to make a referral. They also need an appreciation of what services a referral source can offer before being convinced that a referral is worthwhile for the client. This was demonstrated, in part, by the pattern of referrals we noted. For three BPGs (asthma, delirium-depression-dementia, venous leg ulcers), referrals to sources that were "in-house" (i.e. offered in the institution where the nurses were employed) were more frequent than referrals to external agencies or resources. For the venous leg ulcer BPG, a significant increase in referrals to specialist nurses may indicate an increased likelihood of referrals when there is a clear horizontal referral mechanism for nurses to refer to other nurses.

CRNs were attempting to implement many BPG recommendations in a relatively short time. Recommendations pertaining to referrals may not have been a consistent priority for all sites. We do not know the extent to which CRNs stressed the importance of referrals in their educational sessions. Previous research has demonstrated the importance of training nurses and other professionals on how to make referrals and how to motivate patients to actually keep referral appointments [20,21]. These topics may not have been adequately or consistently addressed during educational sessions, possibly contributing to the lack of positive findings for some BPGs.

Other studies have shown a marked increase in appropriate referrals when this is an explicit and primary intervention focus. For example the introduction of a program aimed at increasing referrals for asthma education of patients consulting at the emergency department for acute asthma found that the number of referrals increased more than 10-fold over four months [21]. Each Asthma Education Centre kept statistics on the number of patients recruited at the emergency department and referred to the center. This program involved education of nurses and respiratory therapists working in the emergency department and in hospital units. Training was focused on a number of key areas including asthma and its treatment, the role of emergency department staff, key messages to provide to patients, services offered at the asthma education centre, and how to make referrals and skills for approaching and motivating patients. The Robichaud et al. (2004) [21] study illustrated the importance of addressing the process of making a referral. This may highlight a weakness of the BPG recommendations used by the implementation sites. The RNAO BPG recommendations describe the importance of referrals and identify the types of referrals that should be made. However, they do not emphasize effective strategies for making referrals such as ways to approach and motivate patients to follow-up on referral recommendations.

Several factors may have contributed to the differential patterns of referrals to clinical services within the agency, referral resources external to the agency and Internet sources. Referrals in-house may have led to positive feedback to the nurses on whether patients were finding the referrals helpful. In part, this may have accounted for the observation that referrals within the agency were more likely to increase than other types of referrals. Nurses may also have been more familiar with in-house referral resources and information about these referral resources may have received more emphasis during education sessions. Nurses and patients alike may not have considered suggestions to seek information on the Internet as a "referral" per se. This may have altered their responses to questions about referrals to the Internet. However, it may also reflect the computer skills of participating nurses and their lack of up-to-date information on credible Internet sites. Nurses have been found to prefer more interactional sources of knowledge compared to e-mails and the Internet [22]. Finally, teams of nurses care for patients. This provides an increased chance of "slippage" if one nurse assumes that another nurse has initiated a referral or discussed a potential agency where the patient can self-refer. Without a formalized means to chart information about the status of patient referrals, the exchange of information about referrals to different resources may be uneven among nurses and between nurses and other health providers.

The focus of this study was on patient referrals that were initiated following introduction of the BPG recommendations. Since making a referral often requires an inter-disciplinary approach, future inquiries should also assess changes in familiarity with referral sources and in reported referrals by other members of the interdisciplinary health care team.

The type of clinical setting where BPG recommendations were implemented may also have influenced referral patterns. Some guidelines are more suited to recommending referrals that would be within nurses' scope of practice. Furthermore, the breastfeeding and smoking cessation guidelines recommend community organizations that are open to self-referrals. However, asthma, venous leg ulcers and delirium-depression-dementia BPGs have a predominance of referral recommendations with a medical focus. Thus, depending on the BPG and the setting, initiating a referral may require a physician's order. However, knowledge of referral sources, increased awareness of the value of appropriate referrals, and familiarity with the means to support patients in following up referrals may all influence effectiveness of the referral process. Differences in intra- and inter-organizational context (e.g. patterns of service delivery within an organization and established inter-organizational partnerships to facilitate referrals), and diverse organizational expectations of nurses' practice (e.g. job descriptions for nurses) may have influenced referral patterns. These factors create different conditions for new referral processes to take hold. However, it is not possible to separate out the relative influence of these contextual factors on referral processes within the participating agencies in this study. Future studies should aim to examine the contribution of these factors on referral processes, thereby providing critical information on the mechanisms by which new referral processes take hold within and between agencies and further guidance on the transferability of findings.

We recommend that health care agencies interested in helping their clients access community resources work collaboratively with agencies in their community to develop strong documentation systems including the use of electronic or web-based formats for referral. While identifying referral sources for inclusion in our tools, CRNs identified many community and Internet-based sources for referral that had been previously untapped by nurses in their setting. Anecdotal evidence through comments of participants indicated that better collaboration between hospital and community sectors is also needed. Prompts on the chart and a rapid web-based or email system would support systematic tracking of referral recommendations and their uptake by patients.

### Limitations

This study examined referrals made by the same nurses prior to and following implementation of the BPGs. Although our study was prospective, there was no concurrent control group of agencies where BPGs were not being implemented. A concurrent control group would be an important addition to future studies to adjust for factors other than BPG implementation that may have changed nurses' referral patterns. In addition, future studies need to have larger numbers of patients for the measurement of longer-term referral outcomes.

The timing of patient interviews may have introduced a recall bias. Response rates for patient interviews were lower than for nurses and thus, patient interview data is likely from a non-representative sample. This is especially likely for the post-implementation patient interviews for smoking cessation and diabetes BPGs. Thus, pre- and post-comparisons of patient reports of referrals should be made cautiously. There was a difference in the nurses' and patients' reports of referral patterns. Since we were not able to match patients to assigned nurses, we cannot determine whether the patients' and nurses' reports of referrals are discrepant, or whether the patients' experiences were with nurses other than those who completed the questionnaire. We did not ask nurses to indicate how many referrals they had actually made in the past month. This might have provided a better gauge of any shift in the nurses' actual referral practices.

A mixed methods study would reveal further insights into what constitutes a referral from the perspectives of patients, families and nurses and how this perspective influences responses to questions about referral practices.

## Conclusion

This study contributes important new information about the impact of implementing nursing BPGs on patient referrals. While nurses' familiarity with referral resources increased significantly for all six BPGs; referrals made by nurses based on their self-reports, data on patient charts and interviews with patients indicated mixed results. Referrals to Internet resources were minimal.

Nurses roles in initiating and supporting appropriate referrals to a variety of providers, agencies and information sources requires further study using mixed methods designs with concurrent control groups. The complementary referral measures we used are promising. BPGs should include specific recommendations on referrals and this information should be tailored to the community setting where implementation is taking place. Nurses can play a vital role in initiating and supporting appropriate patient referrals to a wide spectrum of specialized service providers and to credible information sources such as those available on the Internet. Nurses' familiarity with referral resources does not necessarily change their referral practices. Findings suggest that BPGs should include guidance on effective referral processes. Other patient discharge strategies such as care mapping, the development of clear referral criteria and explicit policies regarding the nurse's role as an initiator of referrals also need to be put in place to reduce the risk of missed referrals and to promote continuity of care.

## Competing interests

The author(s) declare that they have no competing interests.

## Authors' contributions

NE and BD conceived of the evaluation design for this study and developed the nurses' questionnaires. BD led development of the chart audit and patient interview instruments with input from all team members. NE led the psychometric analysis of the referral measures. TV led the teams who developed the best practice guidelines used in the study, and provided overall coordination for the implementation process. JP led the literature review. NE prepared the initial draft of the manuscript. All authors contributed to the interpretation of findings and to writing the final draft of this manuscript. All authors read and approved the final manuscript.

## Pre-publication history

The pre-publication history for this paper can be accessed here:


